# Serum Retinol Levels in Pregnant Adolescents and Their Relationship with Habitual Food Intake, Infection and Obstetric, Nutritional and Socioeconomic Variables

**DOI:** 10.3390/nu8110669

**Published:** 2016-10-25

**Authors:** Laís Spíndola Garcêz, Geania de Sousa Paz Lima, Adriana de Azevedo Paiva, Suzana Maria Rebêlo Sampaio da Paz, Erica Ivana Lázaro Gomes, Valéria Sutti Nunes, Eliana Cotta de Faria, Sílvia de Barros-Mazon

**Affiliations:** 1 Post-Graduate Program in Food and Nutrition, Federal University of Piauí (UFPI), Teresina 64049550, Piauí, Brazil; aapaiva@ufpi.edu.br; 2 Department of Nutrition, Federal University of Piauí (UFPI), Teresina 64049550, Piauí, Brazil; geaniapaz@ufpi.edu.br; 3 St. Augustine College, Nutrition Course, Teresina 64019625, Piauí, Brazil; suzanarspaz@gmail.com; 4 Department of Clinical Pathology, Faculty of Medical Sciences of the State University of Campinas, Campinas 13083887, São Paulo, Brazil; ericaivana@gmail.com (E.I.L.G.); cotta@fcm.unicamp.br (E.C.d.F.); sbmazon@fcm.unicamp.br (S.d.B.-M.); 5 Lipids Lab (LIM10), Endocrinology and Metabolism Division of Hospital das Clinicas, Faculty of Medical Sciences of the University of São Paulo, São Paulo 01246000, São Paulo, Brazil; valeriasutti@gmail.com

**Keywords:** Vitamin A deficiency, vitamin A, pregnancy, pregnancy in adolescence, food consumption, serum retinol

## Abstract

Globally, vitamin A deficiency (VAD) affects about 19.1 million pregnant women. Its occurrence is classically associated with inadequate food intake and may also be associated with socioeconomic factors and the presence of infection. The aim of this study was to determine the factors related to serum retinol levels among pregnant teenagers. The sample consisted of 89 pregnant adolescents, from whom socioeconomic, obstetric, anthropometric, and food consumption data were collected. Serum concentrations of retinol and the supposed presence of infection were determined by high-performance liquid chromatography and C-reactive protein quantification, respectively. The serum retinol concentrations were classified according to the criteria of the World Health Organization. We adopted a 5% significance level for all statistical tests. Serum retinol levels were significantly and positively associated with sanitation (*p* = 0.008) and pre-gestational nutritional status (*p* = 0.002), and negatively with the trimester (*p* = 0.001). The appropriate sanitation conditions and pre-pregnancy body mass index (BMI) were shown to have a protective effect against VAD. Conversely, serum retinol levels were reduced with trimester progression, favoring VAD occurrence.

## 1. Introduction

Vitamin A is an essential micronutrient for all life cycles, especially in times of intense cell proliferation and differentiation, such as in pregnancy. During pregnancy, vitamin A is important for cell division, growth, and maturation of fetal organ and skeletal systems. It is also important for the development and maintenance of the immune and visual systems [1,2,3]. Taking that into consideration, the occurrence of pregnancy during adolescence can be particularly preoccupying, because in addition to the extra supply of nutrients necessary for the development of the fetus, the adolescent mother should receive sufficient and adequate nutrition for her own physical growth and physiological development [4].

Vitamin A deficiency (VAD) is considered a public health problem and is among the main micronutrient deficiencies, present in more than 100 countries, including Brazil [5,6,7]. Globally, VAD affects about 19.1 million pregnant women, and its presence in pregnancy may contribute to infections [8], prematurity [9], anemia [10], and malformations [11], compromising the outcome of pregnancy.

Its occurrence is classically associated with low intake of vitamin A from food sources [5,12], but it may also be associated with other factors such as socioeconomic status, and inadequate sanitation and the presence of infection [13,14,15].

In Brazil, VAD has been documented in the northeast and in some parts of the southeast [16]; however, there are still only a few studies that evaluate the consumption of vitamin A by pregnant women [17], and there are even fewer studies investigating the occurrence of VAD among pregnant teenagers. Furthermore, to our knowledge, no research concomitantly evaluated the association of diet, C-reactive protein levels, and social, nutritional, and gestational variables with the vitamin A status of this population. Therefore, the aim of this study was to investigate the relationship between serum retinol levels and dietary intake, socioeconomic status, obstetrics data, nutrition status and the supposed presence of infection in pregnant adolescents attending a maternity school in northeastern Brazil.

## 2. Methods

### 2.1. Study Characteristics

This was a cross-sectional study evaluating a sample of 89 pregnant adolescents, aged 10–19 years old, who attended a maternity school in Teresina, Piauí, northeastern Brazil. Inclusion criteria were that participants were pregnant adolescents who initiated prenatal care at 20 weeks or less of gestation; that they did not receive vitamin supplements containing vitamin A up to 5 months before conception and during pregnancy; that they were not carriers of disease clinically proven to start pre-pregnancy (i.e., diabetes, liver disease, heart disease, and others), that they were nonsmokers, and that they were experiencing single-fetus pregnancies.

### 2.2. Evaluation of Serum Retinol Levels

Serum concentrations of retinol (dependent variable) were evaluated in a chromatographic AGILENT 1100 coupled to detector diode array (Agilent Technologies, Santa Clara, CA, USA) [18,19]. Blood samples (5 mL) were obtained by peripheral venipuncture and allowed to clot by leaving them undisturbed at room temperature (15–30 min). The clot was removed by centrifugation at 1000–2000× *g* for 10 min in a refrigerated centrifuge, the serum was separated and 200 μL of serum were used for the assay. For the determination of retinol levels, first we performed saponification of the samples, to break ester bonds and obtain retinoids, and then performed an extraction with hexane (Merck), adding 1 mL of saline (0.9% NaCl) and 3 mL of hexane in each tube. The total volume of hexane was then evaporated to dryness in a vacuum samples concentrator, determining the concentration of retinol to the classification of pregnant women as per the cutoffs recommended by Word Health Organization [20], considering the following: deficient/low, retinol <0.35–0.69 mmol/L; acceptable, retinol 0.70–1.049 mmol/L, and normal, retinol ≥1.05 mmol/L. VAD was characterized when retinol levels were <0.70 mmol/L.

### 2.3. Evaluation of Food Intake

The usual dietary intake was evaluated considering the data collected through 24-h recalls (24HR) applied in duplicate. Data from 24HR were used (*n* = 89) and applied at baseline, and 40% were replicated at the second moment (*n* = 36), considering the range of up to 2 months [21,22]. The recall composition analysis was made by NutWin software, version 1.5, of the Department of Health Informatics of the Federal University of São Paulo [23]. We evaluated the intake of vitamin A (µg), zinc (mg/day), and iron (mg/day), with the latter two predicted to be involved in vitamin A bioavailability [24]. The estimated average requirement (EAR) was used as reference value [25]. The person variance of each nutrient was corrected by statistical modeling techniques embedded in the software Multiple Source Method (MSM) (version 1.0.1, 2011, Department of Epidemiology of the German Institute of Human Nutrition Potsdam-Rehbrucke, Nuthetal, Brandenburg, Gemany) [26].

### 2.4. Other Study Variables

Socioeconomic and obstetrical data were obtained through the application of a structured form; these data included age, education level, total household and per capita income, basic sanitation (i.e., water service connected to the public, regular collection garbage, sewage/septic tank), parity, and gestational age.

Schooling was determined in complete years of formal study with approval, by making the following classifications: (1) <8 years of study; and (2) ≥8 years of study. The total family income was divided into minimum wage and defined as the sum of the income of persons with remuneration that occupied the same household; included in this amount were incentives from government programs. The level of per capita income was divided into: (1) up to ¼ minimum wage; (2) more than ¼ to ½ minimum wage; and (3) over ½ to two minimum wages [27].

The sanitation conditions were considered: (1) adequate when there were water services connected to the public network with internal plumbing, regular garbage collection, sewage connected to the public network or existence of a septic tank; and (2) inadequate when one of the sanitation services was absent [28].

Regarding obstetric data, parity was defined as the number of pregnancies that resulted in live or stillbirth, excluding cases of abortion. Gestational age was calculated in weeks from reliable date of the last menstrual period, confirmed by ultrasound, with subsequent confirmation by the method recommended by Capurro et al. [29]. The gestational age classification, at the time of blood collection was given as follows: ≤14 weeks (first trimester) or >14 to ≤20 weeks (second trimester) [30].

Weight and height measurements were obtained with an electronic scale (Welmy model W200/5), with a capacity of 200 kg and 0.050 kg sensitivity, coupled to a metric scale for height measurements with 2.0 limit meters and sensitivity of 1.0 cm. In the evaluation of gestational nutritional status, patients were grouped into three classifications: low weight, adequate, or pre-obesity/obesity. The inclusion of pregnant women in one of these categories was carried out according to the Ministry of Health of Brazil [31], which recommends the classification of body mass index (BMI) by gestational week.

For the classification of pre-gestational nutritional status, BMI/age (BMI/A) was used as proposed by the World Health Organization (WHO) [32]. The cutoff points used to categorize the results were: *z*-score <−2 (low weight); *z*-score ≥−2 to <+1 (adequate); *z*-score ≥+1 <+2 and *z*-score ≥+2 (pre-obesity/obesity).

The inferred presence of infection was defined by the cutoff value equal or higher than (≥)5 mg/L for the acute-phase reactant C-reactive protein (CRP) [33,34]. CRP was determined by Tina-quant CRP (Latex)-HS immunoturbidimetric assay (BM Hitachi 917 Roche, Mannheim, Germany).

Statistical analysis: Data were organized directly in the software database Stata^®^, v.12 (Stata Corp, College Station, TX, USA), where they were analyzed. A descriptive univariate analysis was performed (e.g., frequencies, percentages, measures of central tendency, and dispersion and confidence intervals).

The assumption of normality of the quantitative variables was verified by the Shapiro–Wilk test when necessary, there was log-natural transformation for the normality of the data. The assumption of homogeneity of variances was checked using the Levene test.

To compare serum retinol levels between or among groups in each categorical variable, we used respectively the Mann–Whitney or the Kruskal–Wallis test. To check the correlation between serum retinol levels and quantitative variables of the study, we used the Pearson correlation coefficient. The level of significance in the tests was 5%. The variables with *p* < 0.20 in correlation analyses were included in the multiple linear regression (MLR) model with backward selection. In the final model of MLR, variables with a *p*-value < 0.05 were considered significant. The assumptions for MLR were confirmed in residue analysis.

Ethical aspects: The present study is approved in the Brazil Platform (Opinion Number: 1687138). It is noteworthy that all survey data were preceded by participant confirmation of understanding of the content by informed consent. For participants under the age of 18, this term was also signed by the legal guardians.

## 3. Results

**Figure 1:**
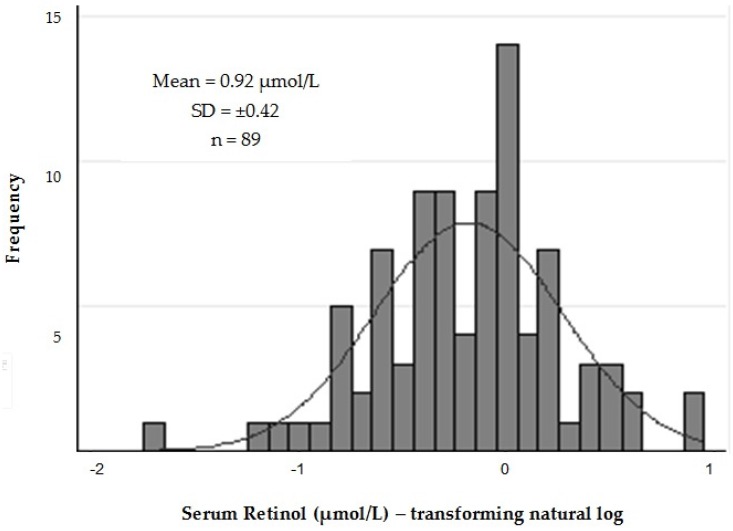
Distribution of serum retinol levels in pregnant adolescents.

Figure 1 shows the distribution of serum retinol levels in study patients after logarithmic transformation (log_10_). The mean (±SD) retinol level found in the study was 0.92 mmol/L (±0.42), and VAD (retinol < 0.70 mmol/L) was diagnosed in 34.8% of pregnant women, indicating the existence of a serious public health problem in this population.

Analyzing the data shown in Table 1, it is observed that in relation to the variables studied, there was a significant difference in serum retinol levels between adequate and inadequate sanitation groups (*p* = 0.033), with the highest average retinol levels in the group having an adequate sanitation service (0.99 mmol/L; CI 95% 0.88–1.11 μmol/L). Also, there was a difference in retinol levels, comparing both moments of blood collection (*p* = 0.002) with the highest mean retinol levels in the group that was in the first trimester of pregnancy (1.01 mmol/L; CI 95% 0.91–1.12 μmol/L).

Analyzing to food intake, significant difference is only observed between the average intake of vitamin A and iron; for vitamin A, there is a higher average consumption in relation to the recommended value for the group and for iron, the average consumption is lower than that recommended (Table 2).

The results of the correlations between serum retinol levels and the different study variables are described in Table 3. Retinol levels were positively correlated with pre-pregnancy BMI (*p* = 0.008), BMI at the time of blood collection (*p* = 0.019) and basic sanitation (*p* = 0.033), and negatively correlated with the trimester at the time of blood collection (*p* = 0.002).

The following variables with *p*-values less than 0.20 in correlation analyses were selected to participate in the MLR model: sewage connected to the public network or existence of septic tank, basic sanitation, trimester at the time of blood collection, pre-gestational nutritional status and nutritional status at the time of blood collection. The model results, obtained by backward stepwise procedure, are presented in Table 4.

**Figure 2:**
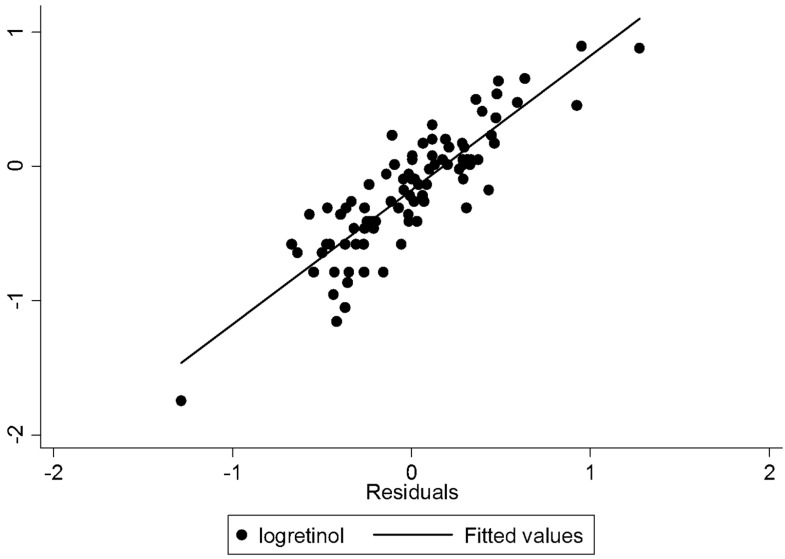
The analysis of residues of the model of multiple linear regression for variable serum retinol levels response * according to the explanatory variables.

Based on the final model of MLR (Model 2), it was confirmed that only basic sanitation, the trimester at the time of blood collection and pre-gestational nutritional status remained significantly related to serum levels of retinol. The existence of a proper sanitation service determined an increase of 0.235 μg/dL (*p* = 0.008) in serum retinol; increasing a trimester reduced serum retinol in 0.239 μg/dL (*p* = 0.001); and each kg/m^2^ more than pre-pregnancy BMI increased serum retinol levels of pregnant adolescents by 0.853 mg/dL (*p* = 0.002). Taken together, these three variables explained 21.3% of the variation in serum retinol levels of the studied population (Table 4). The analysis of residues of MLR (Figure 2) showed that residues were well distributed, indicating homogeneity of variances in behavior and appropriateness of the model.

## 4. Discussion

According to the WHO criteria [20], the frequency of 34.8% of VAD found in this study can be considered a serious public health problem in the target population analyzed. Such a high frequency of VAD differed from the findings of other studies that also investigated its occurrence in pregnant populations. In Iran, Olang et al. [35] found a VAD prevalence of 24.9% among 3270 pregnant women evaluated. In China, a study carried out by Yang et al. [36] that involved 1209 pregnant women showed an inadequate prevalence in only 5.3% of the sample, while in Venezuela, a study by Ortega et al. [37] was the only one with a sample of pregnant adolescents showing a 10.62% prevalence of VAD.

Turning our eyes to Brazilian studies, we realize the impossibility of establishing appropriate comparisons due to the lack of data regarding pregnant adolescents. Thus, despite analyzing adult pregnant women, a study conducted by Fernandes et al. [38], which employed the same cutoff point to assess VAD and also took place in northeast Brazil (at the city of Recife), found a VAD prevalence of 23.1%, which is still lower than the prevalence found in our study.

The low serum retinol levels may be associated with biological, socioeconomic, environmental and anthropometric factors. Accordingly, Miglioli et al. [39] emphasized that VAD presents a clear socioeconomic bias that still prevails in countries, regions, and disadvantaged families, and is related to income, education, housing, and access to health services. Therefore, it is important to emphasize that the different VAD prevalence rates found among the studies should be understood in the context of the factors that are decisive for the decrease of serum retinol in pregnant women in each one of these locations.

Regarding socioeconomic conditions, in this study it was observed that most of the women had access to water supply, sewage collection, and proper waste disposal, resulting in suitable sanitation. This showed a positive linear correlation with retinol levels. According to Aktar et al. [14], this relation is expected, because appropriate sanitation reduces the occurrence of infections, thus exerting a protective effect against VAD. In addition, the authors pointed out that improved sanitation conditions are generally associated with better access to health services, which also has a positive effect on retinol levels.

CRP levels ≥5 mg/L were present in 82% of the population studied, but this result was not correlated with retinol levels. At this point it is important to remember that CRP is not a specific marker of infection; more than that, as an acute phase protein, it is primarily part of the innate immune response and its increase can occur in many different conditions. In fact, CRP levels above normal ranges are recognized as a common state in pregnancy, reflecting the adaptations that occur in maternal immunity and which are necessary for the support of the unborn child. Thus, CRP increase is natural in a healthy pregnancy, rising with advancing gestational age [34,40].

Regarding obstetric variables, there was a negative linear correlation between gestational age and serum retinol levels, which is a similar result to those found in other studies [35,36,37]. It is important to note that even if the reduction occurs with more intensity in the second and third quarter due to plasma volume expansion and increased vitamin A transfer from the mother to the fetus, special attention throughout pregnancy is necessary, particularly in the first trimester, when the major changes in the fetal development occur.

There is a clear scarcity of studies correlating levels of serum retinol and nutritional status of pregnant adolescents. In our study, serum retinol levels showed positive correlation with both pre-pregnancy BMI and BMI at the time of blood collection, but only pre-pregnancy BMI remained significantly associated after MLR analysis. Conversely, Andert et al. [41], in a study with 94 pregnant women in Thailand, showed a positive correlation between gestational BMI and serum retinol levels, but no association between retinol levels and pre-pregnancy nutritional status. However, that study analyzed pregnant women older than 18, and the cutoffs for BMI categorization were different from those used for adolescents. Still, the results obtained by the authors are important, because they reinforce the hypothesis that low weight in pregnancy is related to a low food intake, which reflects negatively on retinol levels. This can also be verified in the study of Pena et al. [42], where the authors assessed the nutritional status of 75 pregnant adolescents in Venezuela, using the same cutoff points as in our study and found low weight in 34.6% of the sample, which also presented insufficient intake of energy, protein, zinc, calcium, and vitamin A.

With regard to food consumption, there was no significant correlation between any of the investigated nutrients and retinol serum levels, which can possibly be explained by the small sample size as well as the absence of large variability among the group.

Divergent results of this study were found by Williams, Eka, and Essien [43], who assessed 101 pregnant women attending a clinic at the University of Calabar, Nigeria. The results showed a significant linear correlation between maternal intake of vitamin A obtained by 24HR and serum retinol. Similarly, Gebreselassie, Gase, and Deressa [44] investigated the relationship between serum retinol levels and food intake of 700 pregnant women in Ethiopia, and also found a positive association between the consumption of foods from animal sources (which provide preformed nutrients) and retinol levels.

However, it is important to highlight that in this study the application of the assessment tool of food consumption was carried out by trained nutritionists and the possible limitations of the method were strictly controlled. Moreover, the replication of the method and the correction of intrapersonal variance of each nutrient provide appropriate information of the usual intake of populations [45], which ensures the reliability of the results obtained.

## 5. Conclusions

Our results showed that VAD is a serious public health problem evaluated in this population, leading to significant and preventable risks in the course of pregnancy, the pregnant adolescent and fetal development. Adequate sanitation conditions have been shown to have a protective effect against VAD, as well as the highest pre-pregnancy BMI. Conversely, increase in the trimester resulted in a reduction in serum retinol, favoring the occurrence of VAD. More research is needed to examine the factors related to the occurrence of VAD in pregnant adolescents, which could serve as a basis for the development of nutritional intervention strategies directed to this population. Moreover, considering the high prevalence of VAD observed in this study, as well as its impact on the pregnancy process, there is a need for regular assessment of the nutritional status of prenatal vitamin A in teens in order to achieve early diagnosis and proper management of this nutritional deficiency.

## Figures and Tables

**Table 1:** Serum retinol levels of the participants according to socioeconomic, obstetric, nutritional, and the presence of infection (CRP) variables.

Variables	*N* (%)	Mean (CI 95%)	Median	*p*-Value
Socioeconomic				
Age (years)				
≤14	11 (12.4)	0.73 (0.60–0.86)	0.70	
>14 to ≤16	40 (44.9)	0.87 (0.74–1.00)	0.87	0.115 *
>16	38 (42.7)	1.02 (0.87–1.18)	0.93	
Schooling (years)				
<8	21 (23.6)	0.79 (0.63–0.95)	0.73	0.091 **
≥8	68 (76.4)	0.96 (0.86–1.06)	0.91	
Per capita income				
≤0.25 SM	17 (19.1)	1.07 (0.86–1.30)	1.01	
>0.25 to ≤0.5 SM	47 (52.8)	0.86 (0.74–0.98)	0.77	0.109 *
>0.5 SM	25 (28.1)	0.93 (0.75–1.10)	0.80	
Water supply				
Public with internal connections	84 (94.4)	1.03 (0.36–1.69)	0.91	0.701 **
Public without indoor plumbing and others	5 (5.6)	0.91 (0.82–1.00)	0.86	
Garbage collection				
Regular	70 (78.6)	0.93 (0.82–1.04)	0.86	0.888 **
Irregular	19 (21.4)	0.88 (0.73–1.04)	0.87	
Sewer connect to the public network or existence of septic tank				
Yes	54 (60.7)	0.96 (0.85–1.08)	0.91	0.134 **
No	35 (39.3)	0.89 (0.76–1.02)	0.79	
Basic sanitation				
Adequate	52 (58.4)	0.99 (0.88–1.11)	0.91	0.033 **
Inadequate	37 (41.6)	0.87 (0.74–0.99)	0.77	
Obstetric				
Parity (number of pregnancies)				
1	77 (86.5)	0.93 (0.84–1.02)	0.87	0.330 **
≥2	12 (13.5)	0.87 (0.53–1.20)	0.66	
Trimester at the time of blood collection				
First (≤14 weeks)	46 (51.7)	1.01 (0.91–1.12)	0.96	0.002 **
Second (>14 to ≤20 weeks)	43 (48.3)	0.82 (0.68–0.96)	0.73	
Nutritional				
Pre-gestational nutritional status (BMI/age)				
Low weight	73 (82.0)	0.92 (0.83–1.01)	0.87	
Adequate	05 (5.6)	0.57 (0.31–0.82)	0.45	0.063 *
Pre-Obesity/Obesity	11 (12.4)	1.06 (0.67–1.45)	1.05	
Gestational nutritional status (BMI)				
Low weight	50 (56.2)	0.95 (0.83–1.07)	0.87	
Adequate	30 (37.7)	0.84 (0.71–0.97)	0.82	0.466 *
Pre-Obesity/Obesity	09 (10.1)	1.04 (0.58–1.49)	0.94	
Infection (CRP ≥ 5 mg/L)				
Yes	73 (82.0)	0.90 (0.81–1.00)	0.84	0.716 **
No	16 (18.0)	0.99 (0.72–1.26)	0.90	

BMI, body mass index (kg/m^2^); per capita income determined by the Brazilian minimum wage (SM) current Brazilian at the study’s time and classified into 03 groups: ≤0.25 MW (≤US $ 48.4), >0.25 to ≤0.5 SM (US $ >48.4 to ≤96.8), ≥0.5 SM (≥US $ 96.8), where 1SAM = US $ 193.58; * Kruskal-Wallis test; ** Mann–Whitney test; CRP, C-reactive protein.

**Table 2:** Comparison of estimated average requirement (EAR) * and average nutrient intake of participants.

Nutrient	Mean (±SD)
Vitamin A (µg)	636.4 (±240.9) **
Estimated Average Requirement (µg/day)	530.0 (±0.0)
Zinc (mg)	10.5 (±3.2)
Estimated Average Requirement (mg/day)	10.5 (±0.0)
Iron (mg)	14.10 (±5.0) **
Estimated Average Requirement (mg/day)	23.0 (±0.0)

* EAR values recommended by the Institute of Medicine [25]; ** Significant difference with the EAR value (*p* < 0.05).

**Table 3:** Pearson’s correlation coefficient of serum retinol levels * with different variables.

Variables	R	*p*-Value **
Socioeconomic		
Age (years)	0.125	0.243
Schooling (years)	0.118	0.267
Per capita income (real)	−0.080	0.441
Water supply	0.061	0.565
Garbage collection	0.008	0.935
Sewer connect to the public network or existence of septic tank	0.162	0.128
Basic sanitation	0.226	0.033
Obstetric		
Parity (number of pregnancies)	−0.125	0.243
Trimester at the time of blood collection	−0.317	0.002
Nutritional		
Pre-gestational nutritional status (BMI/age) *	0.276	0.008
Gestational nutritional status (BMI) *	0.246	0.019
Infection (CRP ≥ 5 mg/L)	−0.068	0.527
Nutrient intake		
Vitamin A (µg) *	0.046	0.666
Zinc (mg) *	0.048	0.654
Iron (mg) *	0.001	0.986

* Natural log format; ** Pearson correlation; BMI, Body Mass Index (kg/m^2^); CRP, C-reactive protein.

**Table 4:** Models of multiple linear regression for variable serum retinol levels response * according to the explanatory variables.

	Coefficient	Standard Error	CI 95%		*p* **
Model 1 **					
Sewer connect to the public network or existence of septic tank	−0.079	0.151	−0.379	0.221	0.603
Basic sanitation	0.300	0.149	0.003	0.597	0.048
Trimester at the time of blood collection	−0.296	0.087	−0.469	-0.123	0.001
Pre-gestacional nutritional status (BMI/age) *	1.275	0.620	0.042	2.509	0.043
Gestational nutritional status (BMI) *	−0.542	0.699	−1.933	0.849	0.441
Model 2 ***					
Basic sanitation	0.235	0.086	0.064	0.406	0.008
Trimester at the time of blood collection	−0.279	0.084	−0.447	−0.111	0.001
Pre-gestacional nutritional status (BMI/age) *	0.853	0.273	0.311	1.396	0.002

* Natural log format; ** **Model 1**: *R*^2^ = 0.248; Adjusted *R*^2^ = 0.202; *** **Model 2**: *R*^2^ = 0.240; Adjusted *R*^2^ = 0.213; BMI = Body Mass Index (kg/m^2^).
